# Supplementary material for: Variation in mental health-related sickness absence duration: The role of occupational health professionals
Source: PLoS One. 2026 May 5;21(5):e0348115. doi: 10.1371/journal.pone.0348115 (PMC13143108; doi:10.1371/journal.pone.0348115)
Supplement: Attachment — Submitted filename: response_reviewers.pdf [file pone.0348115.s002.pdf]

Response letter to the editor and reviewers on the paper 'Variation in Mental Health-Related Sickness Absence Duration: The Role of Occupational Health Professionals'

Sheila Timp, Nicky van Foreest, Willem van Rhenen

2025:08:29

We thank the reviewers and editor for their constructive feedback which helped to improve the content, statements, and quality of the paper. Below we discuss how we dealt with these suggestions and hope to have met their expectations with our new version of the paper.

For the convenience of the editor and reviewers we include their comments in *italics* and provide our reaction in normal font.

General changes

When we began this research, our primary objective was to determine whether differences exist between OHPs. Knowing that large differences in absence duration exist between sectors, we chose to use only data from the education sector, assuming this would minimize the influence of the sectors. Furthermore, we included organization random effects to obtain a more reliable estimate of the variance attributable to OHPs. To place the variance between OHPs in context, we decided to compare the OHP variance with the organizational variance.

The comments of the reviewers and editor made us aware of two problems. First, we expanded our focus from examining *solely* OHPs to examining *both* OHPs and organizations (the initial title of the manuscript also mentioned both OHPs and organizations). However, our literature review remained focused on OHPs and neglected the organizational literature. Second, we overgeneralized our findings by transferring conclusions from one sector to other sectors. Based on this feedback, we decided to substantially revise our study design, thereby hopefully addressing the feedback as provided by the editor and reviewers.

Given the extent of the changes, we think it is best to just summarize the major changes we made, instead of making a (long) list of editorial changes necessitated by these major changes.

1. We now place a primary emphasis on just the variation between OHPs, and treat organizational effects as confounders.
2. We no longer mention the organization in the title of the paper.
3. We include sector as a fixed effect in the model, thereby expanding the analysis from one sector (education) to 19 sectors across the Dutch economy. Table 1 in the revised manuscript provides an overview of these 19 sectors. The updated analysis revealed that organizational

variance (0.10) nearly matches the OHP variance (0.13), demonstrating that our original conclusions could not support recommendations about sector-wide interventions.

4. In the literature section we added references to well-known models (Effort-Reward-Imbalance and Job-Demands-Resources model). However, developing a comprehensive theoretical framework for all return-to-work determinants, we feel, is beyond the scope of this study. In the updated version of the paper we focus specifically on quantifying OHP-level variation using multilevel modeling techniques.

5. We have revised the conclusion according to our new results, see the Discussion section. In the updated manuscript, we conclude that OHPs still have considerable influence on recovery outcomes, while organizational and sectoral effects are also substantial.

6. We added diagnostic categories for mental disorders to control for differences in return-to-work outcomes.

7. We changed our data collection period from 2016–2020 to 2014–2018 to minimize effects of the COVID pandemic.

Response to the Academic Editor

1. *In summary, while the manuscript addresses a relevant topic in occupational health and is based on a robust dataset, several critical issues raised by Reviewer 1 must be carefully addressed before the work can be reconsidered for publication. Most notably, the main conclusion - suggesting that interventions should focus primarily on enhancing OHP practices rather than on organizational reforms - does not appear to be sufficiently supported by the data presented. This inference appears overstated, particularly given that the study design deliberately restricted organizational-level variation by including only organizations within the education sector.* Please refer to the general changes mentioned above.

2. *It seems to me that the greater variation observed at the OHP level is to be expected in this context and does not, in itself, justify prioritizing provider-focused interventions over organizational ones.* Please refer to the general changes mentioned above.

3. *Additionally, the manuscript does not investigate or describe specific practices or characteristics of OHPs that could explain the observed differences in outcomes, limiting the practical utility of this recommendation.* With this paper we actually hope to make a case that a further study into such differences would be very interesting. The aim of the present paper is just to demonstrate that indeed substantial variation between OHPs exists, even after controlling for organizational and sectoral factors.

4. *I would also like to reinforce the reviewer's concern regarding the lack of clarity about what constitutes an "organization" in the analysis. Although organizations are a central comparative unit in the study, the manuscript does not provide a clear definition of what constitutes an "organization". It appears to refer to the employer of the employee, likely individual schools or educational institutions, but this is not explicitly stated.* We added the following information to Section 3.1: "An organization is defined as a company or institution that has a contract with the occupational health service (OHS), where all employees receive occupational health

care from the same OHS. In our dataset, organizations vary considerably in size, with most having at least one thousand employees. The exact number of employees in each organization is unknown to the OHS due to Dutch privacy regulations. Our OHS focuses on on clients from the public sector and large corporate organizations, such as hospitals, factories, municipalities, telecommunications companies, and retail chains."

5. *Furthermore, no details are provided regarding the type (e.g., primary, secondary, higher education), size, or structural features of these organizations. This lack of specificity weakens the interpretation of "organizational-level effects" and limits the reader's ability to assess the plausibility and relevance of the comparisons made.* In the revised study, we extended the analysis to include 19 sectors rather than focusing solely on education. Given this broader scope, we believe the concern about missing details on organizational type, size, or structural features is less central to the interpretation of organizational-level effects.

Reaction to Reviewer 1

1. *The authors make conclusions that are not strongly supported by their data. In particular, they state that their "findings suggest that interventions aiming to improve sickness absence management should focus primarily on enhancing OHP practices rather than on organizational reforms." However, they studied a very narrow slice of organizations and specifically tried to reduce the amount of variation between organizations. As stated by the authors "Furthermore, we minimized variation at other levels by selecting employees working in the same sector in the same country, which strengthens the reliability of the result." Given that the authors explicitly sought to minimize variation at the organizational level in their analyses, it is not surprising that their results show greater variation at the provider level than at the organizational level. It is likely that variation at the level of the organization would be much larger if data were not restricted only to organizations in the educational sector. This design is useful to show that there is large variation in sickness absence at the level of the provider within one employment sector. However, concluding broadly that organizational level interventions should be ignored in favor of provider level interventions is a serious over-generalization of their findings.* Please refer to the general changes mentioned above.

2. *The literature review is rather sparse and some of the citations were puzzling. For instance, citation #4 was cited in conjunction with this sentence "At the job level, several studies have investigated work-related factors and employer characteristics". However, the goal of this paper was to determine "whether adherence to the Dutch occupational mental health guideline by occupational physicians was associated with time to return to work in workers sick-listed due to common mental disorders" - i.e. the paper focused on provider behavior not on employer organizational characteristics. Largely ignored by the authors is the rather large literature demonstrating variations in the incidence/prevalence of mental health issues between workers in different industries, and the role of organizational effects and organizational level interventions to promote mental health among working populations.* Please refer to the general changes mentioned above. We corrected the incorrect citations for work-related factors (see refs. 7-10 in the revised version). We extended the introduction with literature about organizational and sectoral factors, adding citations about organizational-level influences (including organizational size effects and management practices, see refs. 11-13), sectoral influences (private versus public sector, high absence in sectors Health and Education, see ref. 14,15), and theoretical models that combine several levels (job demands resources model and effort-reward imbalance model, refs. 18 and 19). Since we changed the primary objective of the study from examining both OHP and organizational influences to specifically estimating variations among occupational health professionals (OHPs), with organizational factors serving as confounding variables, our literature emphasizes the role of the physician and multilevel approaches.

3. *This paper focuses narrowly on the duration of sickness absence (what happens after you take time off from work for a mental health disorder). It would also be important to report if there was variation in the proportion of workers who took sickness absence between organizations, not just the duration of illness. Given the complexity of return-to-work, this research would benefit from a robust theoretical framework to conceptualize the determinants of RTW, as well as better alignment between observational findings and policy recommendations. The*

study addresses an important question but requires substantial revision given the narrow slice of employers that were studied. For our research question examining differences between OHPs, duration analysis seems appropriate as it measures the effectiveness of return-to-work interventions implemented by OHPs. It would indeed be very interesting to also study absence frequency. However, Dutch privacy regulations prevent occupational health services from accessing data on non-sick-listed employees, making absence frequency unknown for most organizations.

4. *There are some analytic points that could be better explained. The authors provided complete and exhaustive descriptions of the variation in sick leave duration by provider and by organization. As noted above, they unfortunately restricted their analysis to one employment sector, limiting their ability to support their broader conclusions about the relative importance of organization level factors vs. provider factors.* Please refer to the general changes mentioned above.

5. *Another ambiguity was noted. When an employee had multiple periods of absence, each period was included in the dataset as a single case. It is not clear to the reader if one individual could contribute multiple periods to the data (and if so, were these absences treated as independent or clustered events?)* We now account for within-individual clustering using individual-level random effects in our cross-classified multilevel model, treating multiple absence periods from the same employee as correlated rather than independent, see Eq.(1).

6. *The reader does not know what diagnoses or types of mental health disorders included in the cases analyzed. It is likely that different OHPs saw different mixes of cases, which might have different expected recovery times. Variation in OHP performance might be in part attributable to differences in the types of mental health conditions that they saw.* We now include diagnosis as a fixed effect in all models, controlling for 7 main classes of mental health disorders as described in the Dataset Section 3.1.

7. *8. The authors' decision to include data from 2016 to December of 2020 data to avoid COVID-19 effects is questionable, as the pandemic's psychological and workplace impacts likely began influencing mental health and absence patterns well before the December 2020 cutoff for their study. Most people would not consider this to represent a "pre-pandemic" sample.* We revised our data collection period to 2014-2018 to ensure that the COVID-19 pandemic had minimal influence on the results, as most absence cases would conclude before the start of the pandemic.

8. *One point is unclear - the authors never defined what they meant by the term "organization." This appears to mean the employers of the workers studied, but could also mean a trade union or other labor organization. It is not clear the type or size of the employers or organizations.* We added the following information to Section 3.1: "An organization is defined as a company or institution that has a contract with the occupational health service (OHS), where all employees receive occupational health care from the same OHS. In our dataset, organizations vary considerably in size, with most having at least one thousand employees. The exact number of employees in each organization is unknown to the OHS due to Dutch privacy regulations. Our OHS focuses on on clients from the public sector and large corporate organizations, such as hospitals, factories, municipalities, telecommunications companies, and retail chains."

9. *The manuscript does not investigate or describe specific practices or characteristics of OHPs that could explain the observed differences in outcomes, limiting the practical utility of this recommendation. A deeper dive into the characteristics of OHPs that predicted variation in absence time would be interesting.* Actually, as the results of this paper demonstrate that indeed substantial variation between OHPs exists, we hope that this paper provides motivation to extensively study such differences between OHPs, thereby improving employee well being, recovery, and their relation to work.

10. *There are a few minor misspellings (cardiopulmonair, Februari).* We corrected the misspellings.

Comments from Reviewer 2:

1. *Which model parameter estimation method was used: Maximum Likelihood or Monte Carlo?* We used Maximum Likelihood estimation.

2. *Which function from the `lme4` package was used? And what were the arguments adopted in this function?* For Model A (fixed effects only), we used the R function `glm`. For the other models, we used the `glmer` function from the `lme4` package. We now include this information in the Methods section 3.1: "For all four models, we use R (version 4.4.2) with the packages `survival` (version 3.8.3), `lme4` (version 1.1.35.5) and `ggplot2` (version 3.5.1). Model A (baseline model with fixed effects only) is fitted using the `glm()` function, while Models B-D (including random effects) are fitted using the `glmer()` function." In addition, we include the full source code for the model specification as supplementary material S1 so that all details underlying the computations can be found there.

3. *I would like to see the cross-classification table to be able to assess the degree of cross-hierarchy.* We added Table 2, which presents the cross-classification structure. Among the 280 OHPs and 528 organizations, 152 organizations (29%) are linked to multiple OHPs, and 191 OHPs (68%) are linked to multiple organizations, resulting in 892 unique OHP–organization combinations.

4. *The sentence "...However, applying this model requires that the data follow either a two-level hierarchical structure (with observations nested within a single clustering variable) or strictly nested clusters..." The Cox proportional hazard model does not require a hierarchical structure. This sentence seems inappropriate, or I didn't understand the meaning the authors intended.* We acknowledge that our initial statement was incorrect. The Cox model itself does not require a hierarchical structure, although extensions to cross-classified data are not yet well supported in commonly used software. We have revised Section 3.2 of the Methods section as follows: "In our study, OHPs can work for multiple organizations, and organizations can engage multiple OHPs, creating a cross-classified structure. Although frailty or mixed-effects Cox models can in principle accommodate multilevel data, current software provides limited and computationally demanding options for cross-classified designs. Therefore, we model the survival process as a piece-wise exponential process that we can fit using a generalized linear model. This approach accommodates multiple sources of random variation (e.g., OHP and organization effects) in cross-classified data and is well supported by generalized linear (mixed) models (GLMM) software."
