# Supplementary material for: Variation in mental health-related sickness absence duration: The role of occupational health professionals
Source: PLoS One. 2026 May 5;21(5):e0348115. doi: 10.1371/journal.pone.0348115 (PMC13143108; doi:10.1371/journal.pone.0348115)
Supplement: Attachment — Submitted filename: response_reviewers_auresp_2.pdf [file pone.0348115.s003.pdf]

Response letter to the editor and reviewers on the paper 'Variation in Mental Health-Related Sickness Absence Duration: The Role of Occupational Health Professionals'

Sheila Timp, Nicky van Foreest, Willem van Rhenen

2026-04-02

We thank the reviewers and editor for their constructive feedback which helped to improve the content, statements, and quality of the paper. Below we discuss how we dealt with these suggestions and hope to have met their expectations with our new version of the paper.

Responses to questions

1. Have the authors made all data underlying the findings in their manuscript fully available?

The PLOS Data policy requires authors to make all data underlying the findings described in their manuscript fully available without restriction, with rare exception (please refer to the Data Availability Statement in the manuscript PDF file). The data should be provided as part of the manuscript or its supporting information, or deposited to a public repository. For example, in addition to summary statistics, the data points behind means, medians and variance measures should be available. If there are restrictions on publicly sharing data-e.g. participant privacy or use of data from a third party-those must be specified. Reviewer #1: Yes Reviewer #2: No

The data contain confidential medical and employment information and cannot be shared publicly under the terms of our ethics approval and data-use agreements. Deidentified individual-level data may be available under controlled access from the Arbo Unie Data Team (datateam@arbounie.nl) for researchers who obtain ethics approval, sign a data-use agreement, and complete an approved Data Protection Impact Assessment (DPIA).

Reaction to reviewer #1

Minor issues:

- Clarification needed: In 3.1 Dataset, there appears to be a contradiction regarding the sickness absence period used in the analysis (2 years versus 3 years). Please clarify which time period was actually applied in the analysis.

We now explain the time period more clearly in the data section as follows: "Cases are included if the following criteria are met: the employee is between 18 and 65 years old, the sickness absence duration is between one day and three years... The upper limit of three years reflects that some

employers extend financial coverage beyond the mandatory two-year period, either because return to work is expected or due to more generous sickness absence regulations. For our analysis, we truncated absences lasting between two and three years to two years, since employers are only legally required to cover the first two years of sickness absence."

- References: *We revised references and corrected incorrect references.*

Two minor suggestions for the discussion -

- In this sentence "Third, OHPs in occupational health often have more autonomous decision making authority..." Suggest changing "often have" to "may have" as no data were provided to support the assertion that OHP's have more autonomy than hospital-based physicians. (having practiced in both settings in my country, this was not my experience). *This is indeed more appropriate, as the statement was based on limited experience rather than empirical data.*
- "At the organization level, factors such as HR policies, return-to work procedures, case-management capacity and firm size may play a role." I would suggest adding "industry sector" to this list.

Changed
